# Fellowships: Human Embryonic Stem Cell Research

**Published:** 2005-03

The purpose of this program announcement (PA) is to increase the number of biomedical scientists who are pursuing research involving human embryonic stem cells (HESCs). The recent availability of HESCs for federally funded research affords a unique opportunity for investigators to use these cells to address research questions of interest to the mission of the NIH and its component institutes and centers.

Although HESCs have great potential to yield important information on the fundamental properties of cells and disease processes, remarkably little is known about the properties of HESCs that distinguish them from more differentiated cells. Furthermore, very few scientists have had the opportunity to be trained in their use, or to explore the questions that can be addressed to advantage using HESCs. As a result, only a few postdoctoral fellows and established investigators are currently engaged in HESC research, and more investigators must be trained to accelerate the pace of HESC research. Indeed, the NIH Stem Cell Task Force has identified the paucity of skilled researchers and lack of training environments for career enrichment as an important limiting step in the advancement of HESC research.

This PA specifically aims to encourage applications for F32 individual postdoctoral fellowships from promising candidates with the potential to become productive, independent investigators in HESC-related research. It also aims to encourage applications for F33 senior fellowships from experienced scientists who wish to make major changes in the direction of their research or who wish to broaden their scientific background by acquiring new capabilities in HESC research. Only approved HESC lines listed on the NIH Human Embryonic Stem Cell Registry (http://stemcells.nih.gov/registry/) may be used for research training activities. The application must provide the registry identifying numbers of the HESC lines to be used.

The goal of this fellowship program in HESC research is to train highly skilled research scientists to pursue research problems on the basic characteristics of HESCs and their applications to the study of disease. It is expected that these individuals will acquire expertise in the growth and maintenance of HESC that will enable them to pursue research that includes: 1) establishment of HESCs as model systems to explore the fundamental properties of HESCs and their more differentiated progeny; 2) characterization of the molecular events in the differentiation of specific cell types and tissue lineages; 3) establishment of HESCs as model systems for the study of specific diseases; 4) use of HESCs as a primary cell type in drug discovery; and 5) exploration of the therapeutic potential of HESCs in regenerative medicine.

This funding opportunity will use the Kirschstein-NRSA F32 and F33 individual award mechanisms. As an applicant, you will be solely responsible for planning, directing, and executing the proposed project. The number of awards will depend on the quality of applications and funds available.

Applications must be prepared using the PHS 416-1 forms (rev. 6/2002). The PHS 416-1 is available at http://grants.nih.gov/grants/forms.htm in an interactive format. For further assistance contact GrantsInfo, 301-435-0714, e-mail: GrantsInfo@nih.gov. The complete PA is available online at http://grants.nih.gov/grants/guide/pa-files/PA-05-013.html.

Contact: Marion M. Zatz, Division of Genetics and Developmental Biology, NIGMS, Bldg 45, Rm 2AS.25, 45 Center Dr MSC-6200, Bethesda, MD 20892-6200 USA, 301-594-0943, fax: 301-480-2228, e-mail: zatzm@nigms.nih.gov; Ellen M. Werner, Division of Blood Diseases and Resources, NHLBI, 6701 Rockledge Dr, MSC 7950, Bethesda, MD 20892-7950 USA, 301-435-0061, e-mail: wernere@mail.nih.gov; Steven L. Klein, Developmental Biology, Genetics & Teratology Branch, NICHD, 6100 Executive Blvd, Rm 4B01, Rockville, MD 20852 USA, 301-496-5541, e-mail: sk56d@nih.gov; Daniel A. Sklare, Division of Scientific Programs, NIDCD, Bldg 31, Rm 400C, 6120 Executive Blvd, MSC-7180, Bethesda, MD 20892-7180 USA, 301-496-1804, e-mail: sklared@nidcd.nih.gov; James Hyde, Division of Diabetes, Endocrinology, and Metabolic Diseases, NIDDK, 6707 Democracy Blvd, Rm 609, MSC 5460, Bethesda, MD 20892-5460 USA, 301-594-7692, e-mail: jh486z@nih.gov; Judith Podskalny, Division of Digestive Diseases and Nutrition, NIDDK, 6707 Democracy Blvd, Rm 667, MSC 5450, Bethesda, MD 20892-5450 USA, 301-594-8876, e-mail: jp53s@nih.gov; Terry Rogers Bishop, Division of Kidney, Urologic, and Hematologic Disorders, NIDDK, 6707 Democracy Blvd, Rm 619, MSC 5458, Bethesda, MD 20892-5458 USA, 301-594-7717, e-mail: tb232j@nih.gov; Carol Shreffler, Division of Extramural Research and Training, NIEHS, PO Box 12233, Research Triangle Park, NC 27709 USA, 919-541-1445, e-mail: shreffl1@niehs.nih.gov Reference: PA No. PA-05-013

## Scholarly Works in Biomedicine and Health

The National Library of Medicine (NLM) awards small grants for the preparation of book-length manuscripts and other scholarly works of value to U.S. health professionals, public health officials, biomedical researchers, and historians of the health sciences. Grants are awarded for major critical reviews, state-of-the-art summaries, historical studies, and other useful organizations of knowledge in clinical medicine, public health, biomedical research, and the informatics/information sciences relating to them. The scholarly work may be prepared for publication in print or nonprint media, or both.

Scholars in biomedical fields face competing demands for their time, including requirements for clinical care services, grant-related research, and administrative duties. Scholarly work draws upon original sources that may reside within archives, databases, libraries, or human experts around the world, in many different languages and formats. The work of scholarship—discovery, thoughtful analysis, synthesis, and lucid presentation of findings from such materials—requires protected time and support for incidental costs, including materials, staff assistance, and travel. The NLM Grant for Scholarly Works in Biomedicine and Health is intended to help defray such expenses. NLM Grants for Scholarly Works can be used to support several types of scholarly projects.

*Historical works* include 1) scholarly works in the history or philosophy of medicine, public health and the life sciences, the development of medical research and health services, bioethics, and studies on the interrelationship of medicine and society; and 2) scholarly works in the history or philosophy of health informatics, health information sciences, biomedical communications, and health sciences librarianship.

*Critical reviews* include 1) analytical and comprehensive critical reviews which identify the present status of research and practice in various health-related fields, addressing advances which have been made, problems requiring examination, and emerging trends; and 2) scientifically significant and important symposium or conference proceedings related to U.S. priorities in health care, public health, and biomedical research.

*Research aids* include 1) selected secondary tools in the health sciences, such as biomedical guides, atlases, handbooks, dictionaries, indices, catalogs, directories, and other unique reference materials; and 2) English-language translations of important foreign-language classics or primary materials in the history of medicine.

These NLM grants are designed to support scholarly work on a manuscript, video, or electronic resource that will ultimately be published by a commercial or academic press or similar print or electronic dissemination service that ensures quality and availability of the product. Self-publishing by the author will not normally be considered an appropriate dissemination vehicle.

These NLM grants do not support the following types of projects: 1) production of textbooks, curriculum materials or online learning modules; 2) production of works intended for lay audiences; 3) initial reporting of original scientific research findings, including the initial publication of dissertation research; 4) development of coding systems, ontologies, or vocabularies for computational use; 5) publication of proceedings of annual meetings; 6) production of journals, reprints, other serials, or other costs of publishing such as author page charges; 7) operation of established databases; 8) mass digitization of existing archives or print materials; 9) work judged to have significant commercial viability; or 10) projects of local interest only, or works for which access is restricted to a select group.

Applicants should include a topic or chapter outline of the book or work to be produced as part of the research plan. All applicants are required to provide NLM with a copy of the final published work, once it has been issued. NLM recommends that all hardcopy text sponsored in this program be published on acid-free permanent paper as set forth by the American National Standards Institute Permanence of Paper for Publications and Documents in Libraries and Archives (ANSI/NISO Z39.48- 1992).

This funding opportunity will use the G13 award mechanism. As an applicant, you will be solely responsible for planning, directing, and executing the proposed project. An NLM Publication Grant for Scholarly Works provides up to $50,000 in direct costs per year for one, two, or three years. These grants are not renewable. The NLM anticipates spending approximately $1.4 million per year on new awards in this program. Because the nature and scope of the proposed research will vary from application to application, it is anticipated that the size and duration of each award will also vary.

Non-U.S. citizens may apply for these grants; however, priority is given to U.S. citizens. Usually, awards are made three times per year, in December/January, March/April, and August/September. Start dates are usually the same as the award date. The period of performance is one, two or three years.

This funding opportunity uses the just-in-time budget concepts. It also uses the nonmodular budget format described in the PHS 398 application instructions (available online at http://grants.nih.gov/grants/funding/phs398/phs398.html). A detailed categorical budget for the initial budget period and the entire proposed period of support is to be submitted with the application. Applications must be prepared using the PHS 398 research grant application instructions and forms. Applications must have a Dun and Bradstreet (D&B) Data Universal Numbering System (DUNS) number as the universal identifier when applying for federal grants or cooperative agreements. The D&B number can be obtained by calling 1-866-705-5711 or through the website at http://www.dnb.com. The D&B number should be entered on line 11 of the face page of PHS 398. For further assistance contact GrantsInfo, 301-435-0714, e-mail: GrantsInfo@nih.gov. Application receipt dates are 1 June, 1 November, and 1 February. A letter of intent is not required.

Contact: Valerie Florance, Extramural Programs Division, NLM, Rockledge 1, Suite 301, 6705 Rockledge Dr, Bethesda, MD 20892 USA, 301-594-4882, fax: 301-402-2952, e-mail: floranv@mail.nih.gov. Reference: PAR No. PAR-05-025

## Metals in Medicine

The objective of this program announcement (PA) is to encourage research that bridges the areas of inorganic chemistry and medicine in continuation of PA-01-071. The National Institute of General Medical Science (NIGMS) is joined in this announcement by the NIEHS and the NIH Office of Dietary Supplements (ODS).

The mechanisms by which organisms control transition metal ions and the roles of these metals in cellular regulation and signaling in health and disease are of principal interest. The interactions of synthetic inorganic complexes with living systems and their components are an additional area of interest. These areas are linked by the need to involve researchers having a deep understanding of inorganic chemistry in medically relevant research. Much of the work is expected to involve collaborations including chemists, biologists, and medical researchers. The results will be relevant to understanding the mechanisms of metal handling by biological systems and the basic cellular roles underlying the nutritional requirement for essential metals. It is expected that this research will also contribute to the identification of new targets for drug discovery, diagnostics, and future therapeutic approaches involving metal complexes, although drug development, per se, is not a focus of the program.

A higher-order problem presents itself in understanding how the genome-encoded components and the other molecules are constituted in networks of interacting molecules with particular distributions in time and space. Advances in imaging techniques and analytic methods are beginning to yield copious quantitative and spatial data on specific molecules in biological systems. Knowledge of the network and changes in its components over time, and the local rules by which the individual components distribute material and information, will substantially advance our knowledge.

Studies of metalloenzyme structure and function, mechanisms of action, and inhibition are currently well supported and produce results that are utilized in the design of new diagnostic and therapeutic products. Additional stimulation of this area is not needed. In contrast, work in other areas of bioinorganic chemistry lags behind its potential application to human health. These areas include 1) mechanisms of metal metabolism as well as the roles of metals in regulation of cell function and cell–cell interaction, and 2) basic research toward diagnostic and therapeutic applications of metal complexes and of metal chelators and toward exploiting the unique properties of metals for therapeutic applications. The emphasis of this announcement is on the ions, complexes, and organometallic compounds of the transition metals known as lanthanides and actinides, post-transition metals, and metalloid elements.

### Metal Metabolism and Regulation.

Metal metabolism is emerging as an exciting area of cell biology and a potential area for therapeutic intervention. Normal metal metabolism appears to maintain free metal ion concentrations at a very low level and to deliver metals very selectively to their sites of action, while maintaining tight control over their reactivity. Aberrant metal metabolism contributes to pathological conditions such as Menkes’ disease, Wilson’s disease, and hemochromatosis. Intercepting normal metalation reactions may be a way to control metalloprotein activity. Metals may also be associated with the pathology of protein aggregates such as those formed by prions and in Alzheimer’s disease. Metals have also emerged as important sensors and transducers of information with roles in regulation and neurotransmission.

Areas of interest include 1) improved metal ion sensors to study cellular metal ion concentrations and localization; 2) reagents suitable to manipulate those concentrations; 3) identification and characterization of the macromolecular players and vesicular compartments involved in metal ion homeostasis and metal trafficking; 4) elucidation of the roles of metals in cell regulation, signal transduction, and cell–cell signaling; 5) identification and understanding of mRNAs and metal-, oxygen-, and redox-responsive transcriptional and translational regulators, and their potential as therapeutic targets; 6) elucidation of the mechanistic roles of essential trace elements for which metabolic functions are not yet clearly established; 7) analytical tools that accurately monitor biologically important pools, storage pools, and the chemical speciation of metals; 8) biomarkers of exposure and mechanisms of metal toxicity; 9) biomarkers for variable susceptibility to metal toxicity in the human population; and 10) chelation chemistry that can serve as the foundation for therapies to ameliorate aberrant metal accumulations and the effects of toxic exposures.

### Interactions of Metal Complexes with Living Systems.

The therapeutic application of metal complexes is an underdeveloped area of research. Basic principles to guide the development of metallopharmaceuticals are lacking. Metal-containing agents may offer unique therapeutic opportunities. However, significant obstacles, including potential metal accumulations and toxicities, require further research before the promise of medicinal inorganic chemistry can be realized.

Metal complexes may be useful as research probes of biological function, as intermediary lead compounds in the development of non–metal-containing therapeutics, and as potential diagnostic and therapeutic agents. Opportunities exist to exploit the unique properties of metal complexes, (e.g., hydrolytic and redox activity, Lewis acidity, electrophilicity, valency, geometry, magnetic, spectroscopic, radiochemical properties) to measure and/or alter cellular functions. The actions of these compounds may provide insights that are different from those that can be achieved through other chemical, biochemical, or genetic manipulations. Similarly, the actions of metal complexes in whole living organisms are expected to differ in general from the actions of non–metal-containing agents and may offer unique research, diagnostic, or therapeutic opportunities. Principles are needed for the design of safe metal-containing therapeutics.

Another goal of this program is to utilize the power of inorganic chemistry to provide new knowledge of and new approaches for intervention in biological systems. Still another goal is to improve understanding of the reactions of metal complexes in living systems to improve the specificity of these interactions and gain control over the potential toxicity of synthetic metal complexes. The long-term goal is to establish the basic principles of an inorganic medicinal chemistry that will allow for rational design and screening of potential metallopharmaceuticals in the future.

Areas of interest include 1) reactions of metal complexes with cellular constituents (e.g., DNA, RNA, proteins, lipids, carbohydrates, redox substrates, signaling molecules); 2) reactions of metal complexes within the cellular milieu and *in vivo*; 3) uptake of metal complexes into cells and delivery to specific cellular compartments; 4) interactions of metal complexes with specific enzymes and receptors; 5) mechanisms by which synthetic metal complexes recruit cell cycle, signal transduction, and other metabolic pathways to alter cell functions; and 6) structure–activity relationships for ligand design to control metal complex activity and stability *in vitro* and *in vivo*.

The NIH Metals in Medicine meeting report includes a list of specific research opportunities and challenges. This list is intended to be illustrative, not exhaustive. Investigator-initiated ideas are welcome on any subject that will contribute to the objectives listed in this PA.

Research encouraged by this announcement may utilize any appropriate experimental organisms or model systems. For some problems, interesting discoveries may be found in microorganisms from unusual environments and atypical experimental organisms. For other problems, yeast, common invertebrate and vertebrate model organisms, and human cell/tissue cultures may be appropriate. Investigators considering human clinical trials are strongly encouraged to contact the program staff.

This funding opportunity will use the regular research (R01), exploratory research (R21), and program project (P01) award mechanisms. For a description of the R21 grant mechanism see http://grants.nih.gov/grants/funding/r21.htm. For descriptions of the P01 grant mechanism see http://www.nigms.nih.gov/funding/grntmech.html#b (NIGMS) and http://www.niehs.nih.gov/dert/programs/p01.htm (NIEHS).

This funding opportunity uses just-in-time concepts. It also uses the modular as well as the nonmodular budget formats (see http://grants.nih.gov/grants/funding/modular/modular.htm). Specifically, if you are submitting an application with direct costs in each year of $250,000 or less, use the modular budget format described in the PHS 398 application instructions, available at http://grants1.nih.gov/grants/funding/phs398/phs398.html in an interactive format. Otherwise, follow the instructions for nonmodular research grant applications. For further assistance, contact GrantsInfo at 301-435-0714 (telecommunications for the hearing impaired: TTY 301-451-0088) or by e-mail: GrantsInfo@nih.gov.

Applications must be prepared using the PHS 398 application instructions and forms (rev. 5/2001). Applications must have a Dun & Bradstreet (D&B) Data Universal Numbering System number as the universal identifier when applying for federal grants or cooperative agreements. This number can be obtained by calling 1-866-705-5711 or online at http://www.dnb.com/us/. The D&B number should be entered on line 11 of the face page of the PHS 398 form. Applications must be submitted on or before the receipt date described at http://grants.nih.gov/grants/funding/submissionschedule.htm. The complete version of this PA is available at http://grants.nih.gov/grants/guide/pa-files/PA-05-001.html.

Contact: Peter C. Preusch, Division of Pharmacology, Physiology, and Biological Chemistry, NIGMS, Bldg 45, Rm 2AS.43C, MSC 6200, Bethesda, MD 20892-6200 USA, 301-594-5938, fax: 301-480-2802, e-mail: preuschp@nigms.nih.gov; Claudia Thompson, NIEHS, DERT/OPD/CEMBB, MD EC-21, PO Box 12233, Research Triangle Park, NC 27709 USA, 919-541-4638, fax: 919-541-4606, e-mail: thompso1@niehs.nih.gov; Becky Costello, ODS, NIH, Bldg 31, Rm 1B25, MSC 2086, Bethesda, MD 20892-2086 USA, 301-435-2920, fax: 301-480-1845, e-mail: costellb@od.nih.gov. Reference: PA No. PA-05-001

